# Risk Factors for Renal Impairment in Adult Patients With Short Bowel Syndrome

**DOI:** 10.3389/fnut.2020.618758

**Published:** 2021-01-18

**Authors:** Peng Wang, Jianbo Yang, Yupeng Zhang, Li Zhang, Xuejin Gao, Xinying Wang

**Affiliations:** ^1^Department of General Surgery, Jinling Hospital, Medical School of Nanjing University, Nanjing, China; ^2^Department of General Surgery, The First Affiliated Hospital of USTC, Division of Life Sciences and Medicine, University of Science and Technology of China, Hefei, China

**Keywords:** short bowel syndrome, selective digestive decontamination, risk factors, intestinal failure, renal impairment

## Abstract

Renal impairment is a common complication in patients with intestinal failure that is mostly caused by short bowel syndrome (SBS) and is associated with adverse outcomes that severely affect the quality of life or even survival. The prevalence and risk factors for renal impairment in patients with SBS remain unclarified. Therefore, we aimed to determine the prevalence of renal impairment and identify potential risk factors for renal impairment in adult patients with SBS. We retrospectively identified 199 patients diagnosed with SBS admitted to the Department of General Surgery between January 1, 2012 and January 1, 2019, from a prospectively maintained database. Overall, 56 patients (28.1%) with decreased renal function (eGFR < 90 mL/min/1.73 m^2^). The median duration of SBS was 7 months (IQR, 3–31 months) and the mean eGFR was 103.1 ± 39.4 mL/min/1.73 m^2^. Logistic regression modeling indicated that older age [odds ratio (OR), 1.074; 95% CI, 1.037–1.112, *P* < 0.001], kidney stones (OR, 4.887; 95% CI, 1.753–13.626; *P* = 0.002), decreased length of the small intestine (OR, 0.988; 95% CI, 0.979–0.998; *P* = 0.019), and prolonged duration of SBS (OR, 1.007; 95% CI, 1.001–1.013; *P* = 0.046) were significant risk factors for renal impairment. This is the largest study that has specifically explored the risk factors for renal impairment in a large cohort of adults with SBS. The present study showed that renal function should be closely monitored during treatment, and patients should be given prophylactic interventions if necessary. This retrospective study is a part of clinical study NCT03277014, registered in ClinicalTrials.gov PRS. And the PRS URL is http://register.clinicaltrials.gov.

## Introduction

Short bowel syndrome (SBS) is a rare disease usually caused by the removal of the small intestine due to a variety of underlying diseases, and it accounts for 74.4% of chronic intestinal failure (1). In patients with intestinal failure who are treated with long-term parenteral nutrition (PN) support therapy, impaired kidney function has also been reported (2–5).

Renal injury is a serious life-threatening complication that beginning with renal impairment. Worldwide, increasing numbers of patients are affected by chronic kidney disease (6–8). Two factors that have been reported are important for end-stage renal disease (ESRD): aging and type 2 diabetes mellitus (8). The gold standard for evaluating renal function involves inulin or radiolabeled markers to measure the glomerular filtration rate (GFR) (9, 10). However, considering the cost and complexities of measuring GFR, estimated GFR (eGFR) is widely used in clinical work.

In patients with SBS, studies showed that chronic renal failure (CRF) was related to intestinal failure and long-term PN (3, 11). Infections, dehydration, hypovolemic state, and age are also reported were risk factors for renal impairment in patients with SBS; however, these results were not consistent among studies (2–4). In summary, the risk factors of renal impairment (eGFR < 90 mL/min/1.73 m^2^) in adult patients with SBS remain uncharacterized.

Small intestinal bacterial overgrowth (SIBO) occurs commonly in SBS. A study found that among patients with SBS 63% (27 of 43) of patients had SIBO (12). This means that the gut microbiota was disrupted in patients with SBS. A study showed that in patients with SBS, both fecal and colonic biopsy samples were found to have a high prevalence of Lactobacillus, with an associated depletion of Clostridia and Bacteroidetes (13). The crosstalk between the gut microbiota and the host has attracted considerable attention owing to its involvement in various diseases. Many papers report that chronic kidney disease (CKD) is associated with gut dysbiosis and altered host-microbiota crosstalk (14–16). One paper also suggested that the overgrown bacteria translocated from the gut to the blood, where they contributed to the development of CKD (16). Selective digestive decontamination (SDD) is a treatment for SIBO and the principal method that uses broad-spectrum and other antibiotics including those that cover anaerobic bacteria and antifungals (17–19). However, whether there is a crosstalk between gut flora and renal function in patients with SBS remains unclear.

This study aimed to determine the prevalence of renal impairment in clinical practice and to identify potential risk factors for renal impairment in adult patients with SBS.

## Materials and Methods

### Participants

We retrospectively identified consecutive SBS patients from a prospectively maintained database at the Clinical Nutrition therapy center, Jinling Hospital, from January 1, 2012, to January 1, 2019. The prospective database was maintained to enable the guidance of patients' home nutritional support. We included patients who underwent small bowel resection with the length of remaining small bowel <200 cm and were diagnosed with SBS. Patients were excluded if (1) eGFR < 90 mL/min/m^2^ before SBS diagnosed; (2) their serum creatinine and cystatin C level increased significantly in the last 3 months; (3) their remaining intestinal length and the anatomical structure was not clear; (4) the patients were at the acute stage of SBS (<4 weeks after intestinal resection surgery); (5) age < 18 years. The present study was approved by the Ethics Committee of Jingling Hospital. The ethics committee registration number was 2015ZFYJ-010.

Demographic data at admission were collected from the database, including age, sex, body mass index (BMI), nutritional risk screening 2002 (NRS-2002) score (≥3.0 means nutritional risk) (20), subjective global assessment (SGA) score (A means well-nourished; B means moderately malnourished; C means severely malnourished) (21), diabetes mellitus, hypertension, and serum concentrations of albumin, pre-albumin, creatinine, cystatin C, hemoglobin, and C-reactive protein. Unintentional weight loss in recent months (3–6 months), and underlying diseases were recorded. The intestinal anatomy (including small bowel length, anatomy type, presence of an intact ileocecal valve, and colon-in-continuity) were also recorded. Parenteral nutrition (PN) dependence, whether patients received SDD treatment, presence of nosocomial catheter-related bloodstream infections (CRBSIs) were evaluated in hospitalization for intestinal rehabilitation after SBS diagnosis in our center.

### Definitions

The diagnosis of SBS was made in our clinical nutrition center by two gastroenterologists, three surgeons, and one dietitian. The anatomy of the remaining small bowel was used to classify SBS into type 1 (end-jejunostomy with no colon-in-continuity), type 2 (jejuno-colic anastomosis with partial colon-in-continuity), and type 3 (jejuno-ileal anastomosis with an intact colon). The *duration of SBS* was defined as the time interval between the diagnosis of SBS and the previous hospital visit. *Malnutrition* was retrospectively diagnosed by two clinical nutritional physicians, according to the ESPEN diagnostic criteria of 2015 (22). *Kidney stones* were diagnosed based on abdominal imaging (CT, MRI, or ultrasound).

Creatinine and cystatin C were used to calculate the eGFR, using the CKD-EPI equation (23, 24). According to the KIDGO Guideline for the evaluation and management of chronic kidney disease (CKD) which published in 2012, the prognosis of CKD graded by eGFR categories was divided into five grades (G1, normal or high; G2, mildly decreased; G3a, mildly to moderate decreased; G3b, moderate to severe decreased; G4, severely decreased; G5, kidney failure). Hence, we defined the *Renal impairment* as an eGFR of <90 mL/min/1.73 m^2^ (11, 25). The diagnosis of SIBO in patients with SBS mainly depends on two methods; the culture of duodenal fluid was widely used and was considered the gold standard (26), and the other is the hydrogen breath test (27). Nevertheless, both tests raise several issues regarding repeatability, accuracy, and optimal cut-off values (28–30). For these reasons, there is no consensus regarding the optimal test for SIBO. The management of patients with SIBO should consider gastroenterological, surgical, microbiologic, pharmacologic, nutritional, and metabolic factors (31). So, we developed an SDD treatment process combining the etiology, symptomology, and the results of the fecal smear test to determine whether SIBO was present in patients with SBS. We empirically diagnosed patients with SIBO according to (1) the pathogenesis [proton pump inhibitors used for more than 3 weeks; intestinal motility impaired; intestinal anatomy changed (32, 33)], (2) symptoms reported in the literature [diarrhea/stool smelly, abdominal pain, bloating, weight loss, unexpected plateau during the weaning of PN (12, 34, 35)], and (3) the results of the smear test of fecal bacteria (proportion of bacteria was abnormal, and the appearance of other abnormal bacteria). Patients diagnosed with SIBO were then treated with SDD (oral amikacin/vancomycin/gentamicin with metronidazole and fluconazole three times per day for 3 days).

### Statistical Analysis

Numerical data that were normally distributed were expressed as mean ± standard deviation (SD); others were expressed as median (first-to-third interquartile range). Categorical variables were expressed as numbers and percentages. Binary data were presented as 0 (no) or 1 (yes). Data between groups were compared using Student's *t*-test for normally distributed values and the Mann-Whitney *U*-test was used to compare median values, while Pearson's chi-square test was used for categorical variables. Potential risk factors for renal impairment were evaluated by univariate analysis, and risk factors with *P* < 0.1 were included in multivariate binary logistic stepwise regression analysis. In the multivariate analysis statistical significance was set at two-sided *P* < 0.05. Statistical analyses were performed using SPSS software (version 20.0 Win; SPSS Inc., Chicago, IL, USA).

## Results

### Patient Characteristics

**Figure 1:**
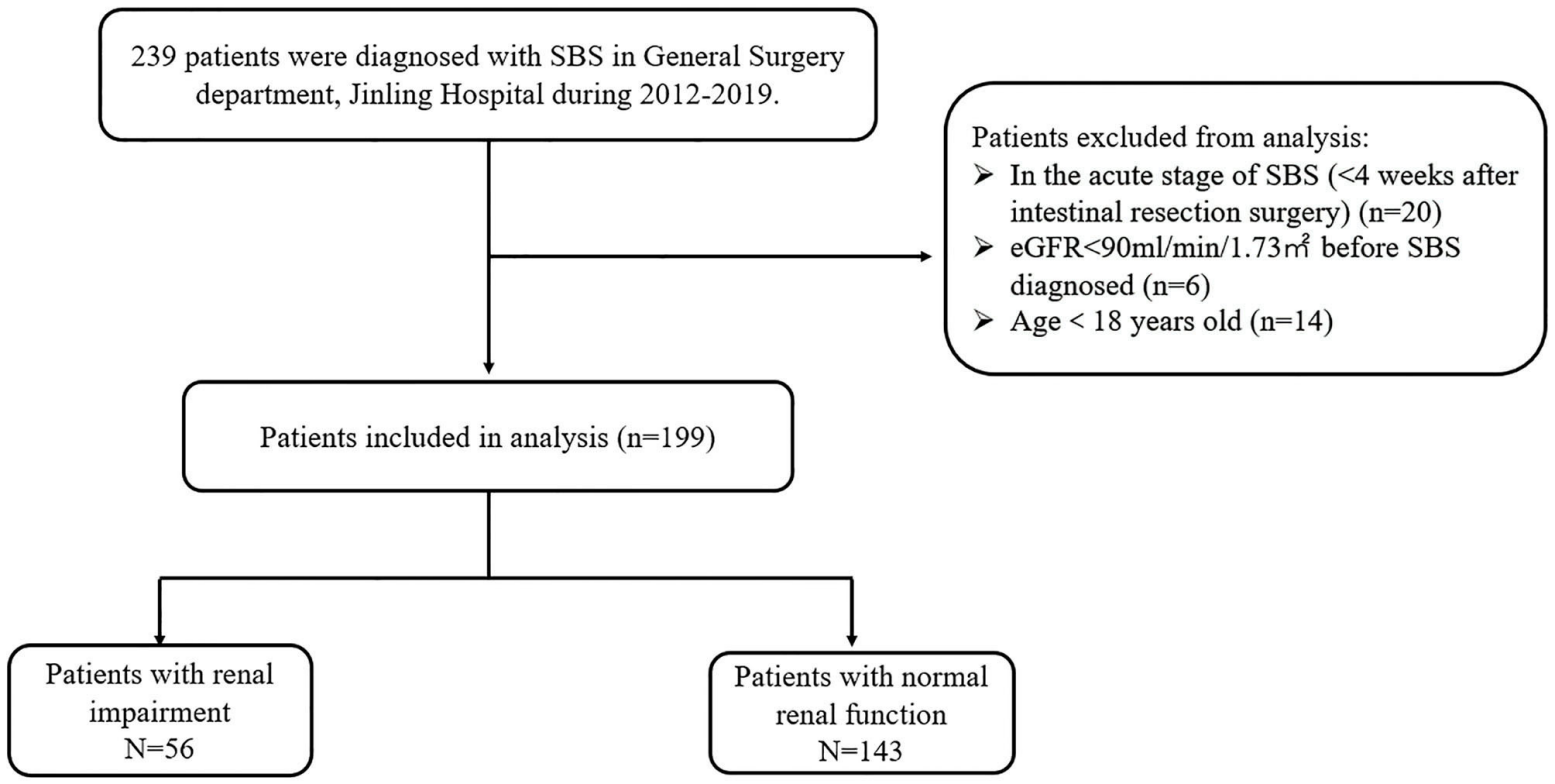
The flow chart demonstrates the selection process of the patients.

A total of 239 patients were diagnosed with SBS in our center during 2012–2019. However, according to our exclusion criteria, data from 40 patients were not used in the present study because they were either in the acute stage of the SBS (20 patients) or eGFR < 90 mL/min/1.73 m^2^ before SBS diagnosed (6 patients) or age < 18 years old (14 patients). Eventually, we identified 199 patients for the study (Figure 1). The mean age was 48.8 ± 17.3 years, and 138 (69.3%) were male. The mean BMI was 18.1 ± 4.6 kg/m^2^. Regarding the SGA score, there were 12 patients in level A (well nourished), 113 patients in level B(moderately malnourished), and 74 patients in level C(severely malnourished), as for the NRS2002 score there are 96 (48.5%) patients with nutritional risk (NRS2002 ≥3). The median duration of SBS was 7 months (interquartile range 3–31). There were 118 (59.2%) patients requiring PN at admission; 141 (71.4%) patients were malnourished according to ESPEN diagnostic criteria of 2015. There were 34 (17.1%) patients with kidney stones; 56 (28.1%) patients with bowel symptoms who received SDD treatment, while 34 (17.1%) contracted nosocomial CRBSIs. The characteristics of the study population at admission are outlined in Table 1.

**Table 1:** Characteristics of adult patients with SBS with and without renal impairment.

**Variables**	**All patients**	Patients with renal impairment^*^	**Patients with normal renal function**	***P*-value**
No. of patients	199	56	143	
Age, year	48.8 ± 17.3	58.6 ± 12.5	44.9 ± 17.4	***<0.001***
Male, *n* (%)	138 (69.3)	43 (76.8)	95 (66.4)	0.154
Diabetes mellitus, *n* (%)	7 (3.5)	1 (1.8)	6 (4.2)	0.682
Hypertension, *n* (%)	23 (11.6)	13 (23.2)	10 (6.9)	***0.001***
Kidney stones, *n* (%)	34 (17.1)	16 (28.6)	18 (12.6)	***0.008***
BMI, kg/m^2^	18.1 ± 4.6	18.0 ± 4.7	18.1 ± 4.6	0.889
Malnutrition, *n* (%)	141 (71.4)	42 (76.8)	99 (69.9)	0.291
NRS2002 ≥ 3, *n* (%)	96 (48.5)	26 (46.4)	70 (49.0)	0.832
<3, *n* (%)	103 (51.5)	30 (53.6)	73 (51.0)	
SGA class (A; B; C)	12/113/74	4/27/25	8/86/49	0.311
Hemoglobin (g/L)	104.5 ± 23.5	97.8 ± 29.6	107.2 ± 20.1	***0.031***
Albumin (g/L)	36.3 ± 6.8	34.7 ± 7.3	36.9 ± 6.6	***0.046***
Serum creatinine (μmol/L)	56.1 (43.0–76.0)	116.0 (89.7–185.5)	50.0 (38.5–61.0)	***<0.001***
Cystatin c (mg/L)	0.8 (0.6–1.0)	1.3 (1.1–1.9)	0.7 (0.6–0.8)	***0.010***
eGFR (mL/min/1.73 m^2^)	103.1 ± 39.4	51.8 ± 27.8	123.2 ± 20.4	***<0.001***
≥ 90, *n* (%)	143 (71.9)	–	143 (100)	
60–89, *n* (%)	25 (12.5)	25 (44.6)	–	
45–59, *n* (%)	10 (5.0)	10 (17.9)	–	
30–44, *n* (%)	6 (3.0)	6 (10.7)	–	
15–29, *n* (%)	6 (3.0)	6 (10.7)	–	
<15, *n* (%)	9 (4.5)	9 (16.1)	–	
ALT (U/L)	33.0 (18.8–58.3)	31.5 (15.8–58)	33.0 (19.0–58.8)	0.547
AST (U/L)	28.5 (18.0–43.0)	25.0 (17.3–59.5)	29.0 (18.0–41.5)	0.124
CRP (mg/L)	3.9 (0.9–19.6)	8.8 (2.0–30.0)	3.0 (0.8–10.9)	0.191
PN requirement, *n* (%)				0.947
Yes	118 (59.2)	33 (58.9)	85 (59.4)	
Weaned	81 (40.7)	23 (41.1)	58 (40.6)	
Duration of SBS, (months)	7.0 (3.0–31.0)	12.0 (5.3–46.0)	5.0 (2.0–16.0)	0.051
SDD, *n* (%)	56 (28.1)	30 (53.5)	26 (18.1)	***<0.001***
Nosocomial CRBSIs, *n* (%)	34 (17.1)	14 (25.0)	20 (13.9)	0.067

*Values were presented as n (%), or mean ± SD, or median (first–to-third interquartile range)*.

*BMI, body mass index; NRS2002, Nutrition Risk Screening 2002; Scr, Serum creatinine; ALT, Alanine aminotransferase; AST, Aspartate aminotransferase; CRP, C-reaction protein; PN, Parenteral Nutrition; SBS, Short Bowel Syndrome; SDD, Selective decontamination of the digestive tract; CRBSIs, catheter-related blood stream infections. Duration of SBS: the time interval between the diagnosis of SBS and the previous hospital visit*.

* *Renal impairment was defined as eGFR <90 mL/min/1.73 m^2^ calculated by the CKD-EPI equation. P-value <0.05 were bolded and italicized*.

The mean small bowel length of the patients was 85.6 ± 45.5 cm; the median was 80.0 cm; 53 (26.6%) patients had type 1, 69 (34.7%) had type 2, and 77 (38.7%) had type 3 SBS. A total of 146 (73.4%) patients had continuity of the colon. The ileocecal valve had been removed in 77 (38.7%) patients (Table 2). The most common underlying diseases were mesenteric ischemia (41.7%), volvulus (23.6%), surgical complications (19.6%), radiation enteritis (6.0%), and others (9.0%) (Table 3).

**Table 2:** The intestinal anatomy of adult patients with SBS with and without renal impairment.

**Variables**	**All patients**	Patients with renal impairment^*^	**Patients with normal renal function**	***P*-value**
No. of patients, *n*	199	56	143	
Small bowel length (cm)	85.6 ± 45.5	75.8 ± 41.2	89.4 ± 46.6	0.068
Median (cm)	80.0 (57.5–117.5)	77.5 (50.0–100.0)	90 (60.0–120.0)	
Anatomy type, *n* (%)				0.962
I	53 (26.6)	13 (23.2)	40 (28.0)	
II	69 (34.7)	23 (41.1)	46 (32.2)	
III	77 (38.7)	20 (35.7)	57 (39.9)	
Ileocecal valve intact, *n* (%)	77 (38.7)	20 (35.7)	57 (39.9)	0.591
Colon in continuity, *n* (%)	146 (73.4)	43 (76.8)	103 (72.0)	0.495

*Values were presented as n (%), or mean ± SD, or median (first-to-third interquartile range)*.

*Type I:end-jejunostomy with no colon-in-continuity*.

*Type II: jejuno-colic anastomosis with partial colon-in-continuity*.

*Type III: jejuno-ileal anastomosis with an intact colon*.

* *Renal impairment was defined as eGFR <90 mL/min/1.73 m^2^ calculated by the CKD-EPI equation*.

**Table 3:** The underlying diseases in adult patients with SBS with and without renal impairment.

**Variables**	**All patients**	Patients with renal impairment^*^	**Patients with normal renal function**	***P*-value**
No. of patients, n	199	56	143	
Mesenteric ischemia	83 (41.7)	30 (53.5)	53 (37.1)	0.266
Radiation enteritis	12 (6.0)	3 (5.4)	9 (6.3)	1.000
Surgical complications	39 (19.6)	9 (16.1)	30 (20.1)	1.000
Volvulus	47 (23.6)	8 (14.3)	39 (27.3)	0.269
Others	18 (9.0)	6 (10.7)	12 (8.4)	1.000

*Values were presented as n (%)*.

* *Renal impairment was defined as eGFR <90 mL/min/1.73 m^2^ calculated by the CKD-EPI equation*.

The mean eGFR of patients with SBS was 103.1 ± 39.4 mL/min/1.73 m^2^. There were 56 (28.1%) patients decreased the eGFR. According KDIGO guideline, in the 56 patients with renal impairment, 25 (44.6%) of them were classified as G2 (eGFR 60–89 mL/min/1.73 m^2^), 10 (17.9%) of them were classified as G3a (eGFR 45–59 mL/min/1.73 m^2^), 6 (10.7%) of them were classified as G3b (eGFR 30–44 mL/min/1.73 m^2^), 6 (10.7%) of them were classified as G4 (eGFR 15–29 mL/min/1.73 m^2^) and 9 (16.1%) of them were classified as G5 (eGFR < 15 mL/min/1.73 m^2^) and four of them required dialysis therapy. The incidence of renal impairment was 23.2% in type 1 SBS, 41.1% in type 2 SBS, and 35.7% in type 3 SBS (*P* = 0.962).

Patients were divided into a renal impairment group (decreased eGFR, eGFR < 90 mL/min/1.73 m^2^) and a normal renal function group (eGFR ≥ 90 mL/min/1.73 m^2^). Compared with patients in the normal renal function group, patients with renal impairment tend to be older (58.6 ± 12.5 years vs. 44.9 ± 17.4 years, *P* < 0.001), having longer duration of SBS (12.0 vs. 5.0 months, *P* = 0.051), and lower hemoglobin concentration (97.8 ± 27.6 vs 107.2 ± 20.1 g/L, *P* = 0.031) and albumin concentration (34.7 ± 7.3 vs 36.9 ± 6.6 g/L, *P* = 0.046). Compared with patients in the normal renal function group, in the renal impairment group there were more patients with kidney stones (28.6 vs. 12.6%, *P* = 0.008), receiving SDD treatment (53.5 vs. 18.1%, *P* < 0.001), with nosocomial CRBSIs (25.0 vs. 13.9%, *P* = 0.067) and hypertensive (23.2 vs. 6.9%, *P* = 0.001). Regarding small bowel length, the renal impairment group was shorter than the other group (75.8 ± 41.2 vs. 89.4 ± 46.6 cm, *P* = 0.068). The two groups were similar regarding sex, BMI, NRS-2002 score, diabetes, PN dependence, and the biochemical indicators of nutritional status (all *P* > 0.1).

### Independent Risk Factors for Renal Impairment in Patients With Short Bowel Syndrome

We incorporated potential variables with *P* < 0.1 in the univariate analysis into a multivariate logistic regression analysis. The results indicated that the age (*P* < 0.001), SBS duration (*P* = 0.046), small bowel length (*P* = 0.019), and existing kidney stones (*P* = 0.002) were independent risk factors for reduced eGFR. SBS duration had an OR of 1.007 per month resulting in a 10-year cumulative risk of 2.3 folds (1.007^120^) (Table 4).

**Table 4:** Multivariate analysis of the factors associated with renal impairment in adults with SBS.

**Variables**	**β**	**OR**	**95%CI**	***P*-value**
Age	0.071	1.074	1.037–1.112	***<0.001***
Kidney stones	1.587	4.887	1.753–13.626	***0.002***
Length of SI	−0.012	0.988	0.979–0.998	***0.019***
Duration of SBS	0.007	1.007	1.001–1.013	***0.046***
Hypertension	0.140	1.150	0.338–3.910	0.823
Hemoglobin	−0.016	0.984	0.961–1.008	0.183
Albumin	0.041	1.042	0.955–1.136	0.441
SDD	0.820	2.272	0.897–5.755	0.356
CRBSIs	−0.088	0.916	0.313–2.679	0.837

*SBS, Short bowel syndrome; SI, Small intestine; SDD, Selective decontamination of the digestive tract*.

*Duration of SBS: the time interval between the diagnosis of SBS and the previous hospital visit. P-value < 0.05 were bolded and italicized*.

## Discussion

To the best of our knowledge, this is the largest study that has specifically explored the risk factors for renal impairment in a large cohort of adults with SBS (*n* = 199). The incidence of renal impairment in adults with SBS was 28.1%, and the median duration from SBS diagnosis to the development of renal impairment was 12 months. Renal impairment in adults with SBS was influenced by age, shorter length of the small bowel, kidney stones, and prolonged duration of SBS.

Renal impairment has been widely reported in patients with SBS. To date, Buchman et al. (3) reported decreased GFR >20% in 52.5% of patients, and 3.5% decline per year of creatinine clearance and a reduced kidney function in ~50% of patients was observed; Lauverjat et al. (4) reported that decreased GFR was found in 9 of 16 patients with SBS also receiving home parenteral nutrition (HPN). Some risk factors have been reported, including infections, dehydration, hypovolemic state, duration of HPN, and patient age; however, these results were not consistent among studies (2–4). Both studies had results similar to ours. Ylinen et al. (11) found that small bowel length was a risk factor for renal impairment in children. Agostini et al. (36) found that age, urologic disease, sepsis, and SBS significantly influenced the development of CKD in patients with HPN, and that eGFR comparable with the median decreased −2.4 to −7.3% per year. Because our study absorbed all patients either receiving PN support therapy, or weaned off PN, and receiving enteral nutrition support therapy, the incidence of renal impairment only was 28.1%.

Patients with SBS need long-term PN. However, with the progress of treatment the amount of fluid and basal metabolism required by patients changed significantly. Most patients do not adjust their PN support plan timeously, resulting in them becoming chronically dehydrated for long periods. A retrospective study of 33 patients with long-term PN found that the GFR decreased by 3.5% ± 6.3% per year (3). Similarly, in our study, we found that the cumulative risk of eGFR was 2.3 fold (1.007^120^) after 10 years. A decrease in renal function with aging is a natural phenomenon.

In patients with SBS, renal impairment is mainly due to systemic fluid imbalance caused by excessive loss of ostomy fluid, CRBSIs, chronic dehydration, and electrolyte imbalance (37). The small bowel length is an important factor that can affect nutrients and electrolyte absorption, as well as renal function (11). Similar to the results of previous reports, the length of the small intestine in our study was an important factor for determining renal impairment. In the present study, nosocomial CRBSIs are not considered independent risk factors for renal impairment because we did not analyze the occurrence of CRBSIs during HPN, and CRBSIs are well-controlled during hospitalization. However, in the renal impairment group, a higher percentage of patients suffered CRBSIs (25.0 vs. 13.9%).

The ileocecal valve plays an important role in the alimentary canal since it can influence nutrients absorbed and keep electrolytes balanced. Although in the present study the renal dysfunction has no difference in patients with or without the ileocecal valve, we found that the eGFR of SBS in various types were significantly different (type 1 SBS was 107 ± 33.5 mL/min/1.73 m^2^, type 2 SBS was 94.1 ± 43.7 mL/min/1.73 m^2^ and type 3 SBS was 108.4 ± 38.1 mL/min/1.73 m^2^, type 2 vs. type 3, *P* = 0.037). The eGFR was significantly lower in patients with type 2 SBS than in type 3 SBS. Although type 1 SBS includes patients with stomas who are prone to water and electrolyte loss, no significant reduction in eGFR was found. This phenomenon may be related to the acute phase of the disease, since most of the acute phase occurs during the hospitalization period when doctors pay more attention to water and electrolyte balance.

Kidney stones have been reported in patients with SBS, resulting in adverse clinical sequelae (38, 39). The prevalence of kidney stones is increased in patients who have undergone intestinal surgery, especially in patients with jejunostomy or ileostomy, with an occurrence of 5–15% (40–42). Patients with kidney stones have significantly increased incidences of adverse renal outcomes, including chronic renal failure (43). Our previous studies also found that patients with kidney stones have lower eGFR than patients without kidney stones (44). In the present study, we found that patients with kidney stones may be prone to renal impairment. Nevertheless, the mechanisms linking nephrolithiasis to impairment of renal function remain unclear. The intestinal surgery alters the anatomy of intestinal results in malabsorption of bile acids and fatty acids (45). Normally, the oxalate in dietary binds to calcium in luminal forming calcium oxalate which cannot be absorbed. At the same time, unabsorbed fatty acids bind to luminal calcium and form insoluble calcium soaps which inhibit the binding of luminal oxalate to calcium and increase its absorption. Besides these, the bile acids enrichment in colon and increases the absorption of oxalate, results in hyperoxalatemia and increase the formation of oxalate stones (38). There is also a study found that the plasma oxalaye increase at lower GFR levels even among those without entric or primary hyperoxaluria and established the relationships between plasma oxalate, GFR and urine oxalate among patients with routine urinary stone disease. In present study, we found that the patients with kidney stones may be prone to renal dysfunction, may be the decreased of GFR resulted the kidney stones (46).

SDD is an infection preventive measure for ICU patients that was proposed more than 30 years ago (47). It has been reported to have a favorable effect on mortality in adult patients in general intensive care units (48). In patients with SBS, a review showed that cyclical use (1 week per month) of broad-spectrum antibiotics was the mainstay of therapy for small-intestinal bacterial overgrowth (49).

We diagnosed patients with SIBO using our SDD management scheme. Many papers reported that disordered gut flora could influence the renal function of the host. Vaziri et al. (50) found that there were 190 bacterial OTUs in the stool, with marked differences in abundance between patients with ESRD and normal controls. In addition, Wang et al. performed a clinical trial that showed that bacterial DNA was detected in the blood of six (20%) patients with ERSD. The bacterial genera found in the blood that were overgrown in the intestines were mainly *Klebsiella* spp., *Proteus spp, Escherichia* spp., *Enterobacter* spp., and *Pseudomonas* spp. (16). This paper suggests that the overgrown bacteria translocated from the gut to the blood, where they contributed to the development of CKD. Hence, SIBO may be an independent risk factor for renal impairment in patients with SBS. However in the present study, 56 (28.1%) patients received SDD treatment (may with SIBO), less than the previously reported value (63%) (12). The reason for our low result is that we can only rely on symptoms and experience to diagnose SIBO. And SIBO is not an independent risk factor for renal impairment in patients with SBS. The relationship between intestinal flora disorder and SBS-related kidney damage needs further study.

The present study had several limitations. First, it was a retrospective single-institution study performed at a tertiary-care referral center. Second, the dietary habits, home PN formulation, and total time that patients required PN support therapy were not available. Third, the incidence of CRBSIs during HPN could not be recorded. Fourth, because most patients with SBS in our center were adults, we only evaluated the risk factors for renal impairment in adults. Fifth, it was a retrospective study performed at a tertiary-care referral center, the data of urine test (hematuresis and proteinuria) was lacking. Therefore, the incidence of renal impairment may have been underestimated. Large, multicenter, prospective studies focusing on the reason for renal impairment in both pediatric and adult patients with SBS should be conducted in the future.

The strengths of the present study. Firstly, it is the largest study that has specifically explored the risk factors for renal dysfunction in a large cohort of adults with SBS (*n* = 199). In addition, we found that the duration of SBS, kidney stones, old age, and the length of the small bowel are the independent risk factors for renal dysfunction in adult patients with SBS. And, SBS duration had an OR of 1.007 per month resulting in a 10-year cumulative risk of 2.3 fold (1.007^120^). Given that patients with SBS have increased risk for renal impairment and associated adverse outcomes, close monitoring of renal function and prophylactic interventions should be conducted routinely in clinical practice.

## Conclusion

Renal function impairment is common in adults with SBS and can results in adverse clinical consequences. In our study, 56 (28.1%) in 199 patients developed renal function impairment with eGFR < 90 mL/min/1.73 m^2^, 34 (17.1%) developed nephrolithiasis. The risk of renal impairment is increased in patients with older age, kidney stones, shorter length of remaining small intestine, and prolonged duration of SBS. In the chronic stage of SBS, renal impairment is primarily related to water and electrolyte loss, kidney stones, old age, and the length of the small bowel. Given that patients with SBS have increased risk for renal impairment and associated adverse outcomes, close monitoring of renal function to avoid dehydration, and prophylactic interventions should be conducted.

## Data Availability Statement

The raw data supporting the conclusions of this article will be made available by the authors, without undue reservation.

## Ethics Statement

The studies involving human participants were reviewed and approved by the Ethics Committee of Jingling Hospital. The patients/participants provided their written informed consent to participate in this study.

## Author Contributions

PW and XW contributed to conception, design of the research, and critically revised the manuscript. PW and JY contributed to acquisition, analysis, and interpretation of the data. XG, LZ, and YZ drafted the manuscript. All authors agreed to be fully accountable for ensuring the integrity and accuracy of the work, read, and approved the final manuscript.

## Conflict of Interest

The authors declare that the research was conducted in the absence of any commercial or financial relationships that could be construed as a potential conflict of interest.
